# The influence of anti-TNF therapy on the magnetic resonance enterographic parameters of Crohn’s disease activity

**DOI:** 10.1007/s00261-015-0466-0

**Published:** 2015-06-07

**Authors:** Piotr Eder, Katarzyna Katulska, Iwona Krela-Kaźmierczak, Kamila Stawczyk-Eder, Katarzyna Klimczak, Aleksandra Szymczak, Krzysztof Linke, Liliana Łykowska-Szuber

**Affiliations:** Department of Gastroenterology, Human Nutrition and Internal Diseases, Heliodor Swiecicki Clinical Hospital, Poznan University of Medical Sciences, Przybyszewskiego Street 49, 60-355 Poznan, Poland; Department of General Radiology, Heliodor Swiecicki Clinical Hospital, Poznan University of Medical Sciences, Przybyszewskiego Street 49, 60-355 Poznan, Poland

**Keywords:** Anti-tumor necrosis factor alpha therapy, Crohn’s disease, Magnetic resonance enterography

## Abstract

**Purpose:**

Magnetic resonance enterography (MRE) is a useful tool in assessing the transmural and extraintestinal lesions in Crohn’s disease (CD). However, the influence of anti-tumor necrosis factor (anti-TNF) therapy on MRE features of CD severity remains unknown. The purpose of the study was to assess the short- and long-term changes in MRE features of CD activity in relation to CD clinical course in patients treated with anti-TNF antibodies.

**Methods:**

The influence on the most important parameters of CD activity seen in MRE was assessed retrospectively using a validated score. Patients were treated with anti-TNF agents and the clinical, laboratory, and MRE CD activity was estimated at baseline, after the induction therapy and after 1 year of treatment.

**Results:**

71 patients were enrolled in a study. The change in CD clinical activity correlated significantly with fluctuations in MRE activity score (*P* < 0.0001, *r* = 0.5 for induction; *P* = 0.004, *r* = 0.7 for maintenance anti-TNF therapy, respectively). Bowel wall thickening, mesenteric lymphadenopathy, and fat wrapping with vascular proliferation were MRE parameters which changed significantly both after the induction and maintenance treatment in patients responding to the therapy. The change in MRE activity score was mostly pronounced during the first 3 months of treatment, when compared to the continuation of the therapy till week 52–54 (−6 points vs. −2 points, respectively; *P* = 0.0008).

**Conclusions:**

Transmural and extraintestinal healing seen in MRE correlates with changes in CD clinical activity during anti-TNF therapy, thus MRE seems to be a useful tool in monitoring the efficacy of biological agents.

In recent years, magnetic resonance enterography (MRE) has become one of the most important diagnostic methods in the assessment of Crohn’s disease (CD) activity [1]. Due to its non-invasiveness, it can be performed multiple times in the same patient, what enables the dynamic estimation of disease progression or regression in time [2].

Anti-tumor necrosis factor alpha (anti-TNF) antibodies are novel potent anti-inflammatory drugs in CD [3]. Nevertheless, it is still a matter of debate, how to monitor the course of anti-TNF therapy. Endoscopy is believed to be a gold standard; however, due to its invasiveness it is questionable to repeat this investigation in a short period of time in all patients [3]. That is why it seems that MRE could be a valuable alternative for endoscopic assessment, and in the case of small bowel CD—even a method of choice for dynamic monitoring of the course of anti-TNF therapy. Moreover, MRE also seems to be the most accurate tool in determining so called transmural healing in CD, as it allows to visualize all layers of the gut wall [4]. However, taking into account a broad spectrum of inflammatory intestinal and extraintestinal CD manifestations in MRE, there are still few data concerning the direct influence of anti-TNF therapy on radiological parameters of CD activity. That is why, due to an increasing role of MRE in diagnostics of CD, there is an urgent need to determine which morphological changes in the inflamed intestines result from anti-TNF therapy. This could help in a better understanding of the mechanisms of the action of biological agents, and it would provide important data on the definition of transmural and extraintestinal healing, as there are still many difficulties in defining this phenomenon. Thus, the main aim of the current study is to assess which radiological features of CD severity in MRE are mostly influenced by anti-TNF antibodies in relation to the efficacy of biological therapy.

## Patients and methods

We retrospectively analysed data from patients with diagnosed CD, who were treated with anti-TNF agents in years 2009–2014 in our department. All patients were treated according to the current therapeutic guidelines with either adalimumab (ADA) or infliximab (IFX) [5]. ADA was administered in the following doses: 160 mg at week 0, 80 mg at week 2, 40 mg every other week till week 12 (7 induction doses), and then in patients responding to induction therapy—40 mg every other week till week 52. IFX was administered in the doses of 5 mg/kg body weight at week 0, 2, 6 (3 induction doses), and then in patients responding to induction therapy—every 8 weeks till week 52. Response to the induction therapy was defined clinically as a decrease in Crohn’s Disease Activity Index (CDAI) by 100 points or more. Clinical remission was defined as CDAI < 150 points [6].

The inclusion criteria were as follows:

1. Qualification for biological therapy because of CD flare (as assessed by CDAI) not responding to standard medical therapy. Standard medical therapy was defined as therapeutic and stable doses of steroids (1 mg of prednisolone/kg body weight) for at least 3 weeks, and/or azathioprine (AZA—2–2.5 mg/kg body weight), and/or mesalamine (4 g daily) for at least 12 weeks.
2. Ileal or ileocolonic CD location.
3. Performance of MRE twice: at baseline—up to 3 weeks before starting anti-TNF therapy, and after induction doses of biological agents: at week 12–14 in the case of ADA and at week 9–12 in the case of IFX. Only MRE investigations performed with the same protocol were taken into account. When possible, also MRE studies performed after 1 year of anti-TNF therapy were analysed.

Exclusion criteria:

1. Isolated colonic CD.
2. Significant changes in therapeutic regimens of anti-TNF therapy defined above or in the concomitant treatment, like for example, the introduction of a new drug, a change in the dose of immunosuppressant. Only tapering of steroids after finishing the induction phase of anti-TNF therapy or discontinuation of antibiotics were allowed in patients responding to the biological treatment.
3. Previous anti-TNF therapy <12 weeks before enrolment to the current analysis.

Clinical activity was assessed by CDAI. The severity of endoscopic lesions was estimated by calculating Simple Endoscopic Score for Crohn’s Disease (SES-CD) [7]. Both SES-CD and CDAI were calculated prospectively. Biochemical activity was assessed in parallel.

MRE studies were performed with the same protocol as described previously [8]. Patients fasted for 6 h before MRE. 30–40 min before scanning, patients were administered 1500 ml of oral polyethylene glycol. Fifteen minutes before the procedure, 40 mg of buscolysin was injected intravenously in order to reduce bowel motility. The study protocol consisted of the following sequences:

1. true fast imaging with steady-state free precession sequences in the coronal and transverse planes,
2. single-shot turbo spin-echo sequences with fat suppression in the coronal plane,
3. cine loop coronal images for the visualization of the bowel movements and stenosis,
4. fat-suppressed 3D T1-weighted Volumetric Interpolated Breath-Hold Examination and T1-weighted 3D Fast Low Angle Shot technique before and three times—30, 90 s, and 5 min—after intravenous injection of gadolinium contrast (dose of 0.1 mmol/kg body weight followed by 20 ml of saline).

All MRE investigations were assessed by a radiologist with more than 12 years of experience in this cross-sectional imaging technique. All studies were blinded for the radiologist and then randomly analysed and quantified using the grading score called the Simple Enterographic Activity Score for Crohn’s Disease (SEAS-CD), whose diagnostic utility was previously proved in an independent cohort of CD patients by Eder et al. [8]. This score consists of the most important features reflecting CD activity, which were quantified depending on their intensity and they formed the final SEAS-CD result (Table 1).

**Table 1:** Simple Enterographic Activity Score for Crohn’s Disease (SEAS-CD) [8] All variables are calculated separately for jejunum and ileum, and in the next step they are summed up

MRE feature	Grading scale		
Bowel wall thickening	<3 mm: **0 pts**	3–7 mm: **1 pt**	>7 mm: **2 pts**
Contrast enhancement	None: **0 pts**	Homogenous pattern: **1 pt**	Layered pattern: **2 pts**
Fat wrapping^a^	None: **0 pts**	Present: **1 pt**	
Proliferation of mesenteric vasculature^a^	None: **0 pts**	<5 vessels/3 cm^2^ of mesenteric fat: **1 pt**	≥5 vessels/3 cm^2^ of mesenteric fat: **2 pts**
Mesenteric lymphadenopathy	None: **0 pts**	<10 enlarged (diameter > 5 mm) lymph nodes: **1 pt**	≥10 enlarged (diameter > 5 mm) lymph nodes: **2 pts**
Bowel wall ulcerations	None: **0 pts**	At least one ulceration present, not exceeding ½ of bowel thickness: **1 pt**	At least one ulceration present, exceeding ½ of bowel thickness: **2 pts**
Stenotic complications	None: **0 pts**	Stenosis without prestenotic dilatation: **1 pt**	At least one stenosis with prestenotic dilatation: **2 pts**
Intra-abdominal fistulas	None: **0 pts**	At least one intra-abdominal fistula tract visible: **5 pts**	
Extent of the disease in jejunum or ileum	<1500 mm: **1 pt**	>1500 mm: **2 pts**	

Scoring of each parameter is presented in bold

^a^Fat wrapping and proliferation of mesenteric vasculature scores were summed up, as those phenomena are strictly interrelated and they are assessed together

As the first step, we determined the influence of induction doses of anti-TNF agents on CD activity assessed in MRE. We compared changes in the most important radiological features of CD activity in patients who responded to the therapy (defined as a decrease in CDAI by 100 points—the responders group) with patients who were primary non-responders. Then the influence of long-term anti-TNF therapy was also analysed by comparing MRE results after induction doses of biological agents with MRE CD activity scores after 1 year (at week 52–54) of treatment.

### Statistical analysis

Data are presented as means with standard deviations (SD). Correlation analyses between selected parameters were performed with the use of Spearman’s rank correlation coefficient. Statistical differences were calculated using Student *t* test (parametric) when conditions of normality and equal variance were met. When the normality test failed, the Wilcoxon test or Mann–Whitney test was used for paired or unpaired groups, respectively. A *P* value < 0.05 was considered significant. All data were analysed using the GraphPad Prism Version 6.0 (GraphPad Software Inc., USA).

## Results

71 CD patients, in whom MRE imaging was performed before and after induction anti-TNF therapy, were enrolled into the study. Baseline characteristics of the whole study group are presented in Table 2.

**Table 2:** Baseline characteristics of the whole study group (*n* = 71). Data are presented as means with standard deviations (SD)

Feature	
Age (years)	30 ± 9
Male/female—*n*	32/39
Simple Enterographic Activity Score for Crohn’s Disease	14 ± 5
Disease duration (years)	5 ± 3
High sensitivity C-reactive protein (mg/l)	19.7 ± 25.3
Erythrocyte sedimentation rate (mm/h)	29 ± 19
Hemoglobin (g/dl)	11.9 ± 2.1
White blood cell count (10^3^/mm^3^)	7.1 ± 3.5
Platelets (10^3^/mm^3^)	367 ± 112
Crohn’s Disease Activity Index	273 ± 89
Simple Endoscopic Score for Crohn’s Disease (*n* = 31)	13 ± 8
Disease location—*n* (%)	
L1 (ileal)	28 (39%)
L3 (ileocolonic)	43 (61%)
Disease behavior—*n* (%)	
B1 (inflammatory)	54 (76%)
B2 (stricturing)	5 (7%)
B3 (penetrating)	12 (17%)
Medications—*n* (%)	
Steroids	57 (80%)
Azathioprine	57 (80%)
Aminosalicylates	68 (96%)
Antibiotics	24 (34%)
Previous anti-TNF therapy	9 (13%)
Anti-TNF agent used: adalimumab/infliximab—*n* (%)	28/43 (39%/61%)

### Anti-TNF induction therapy

**Fig. 1:**
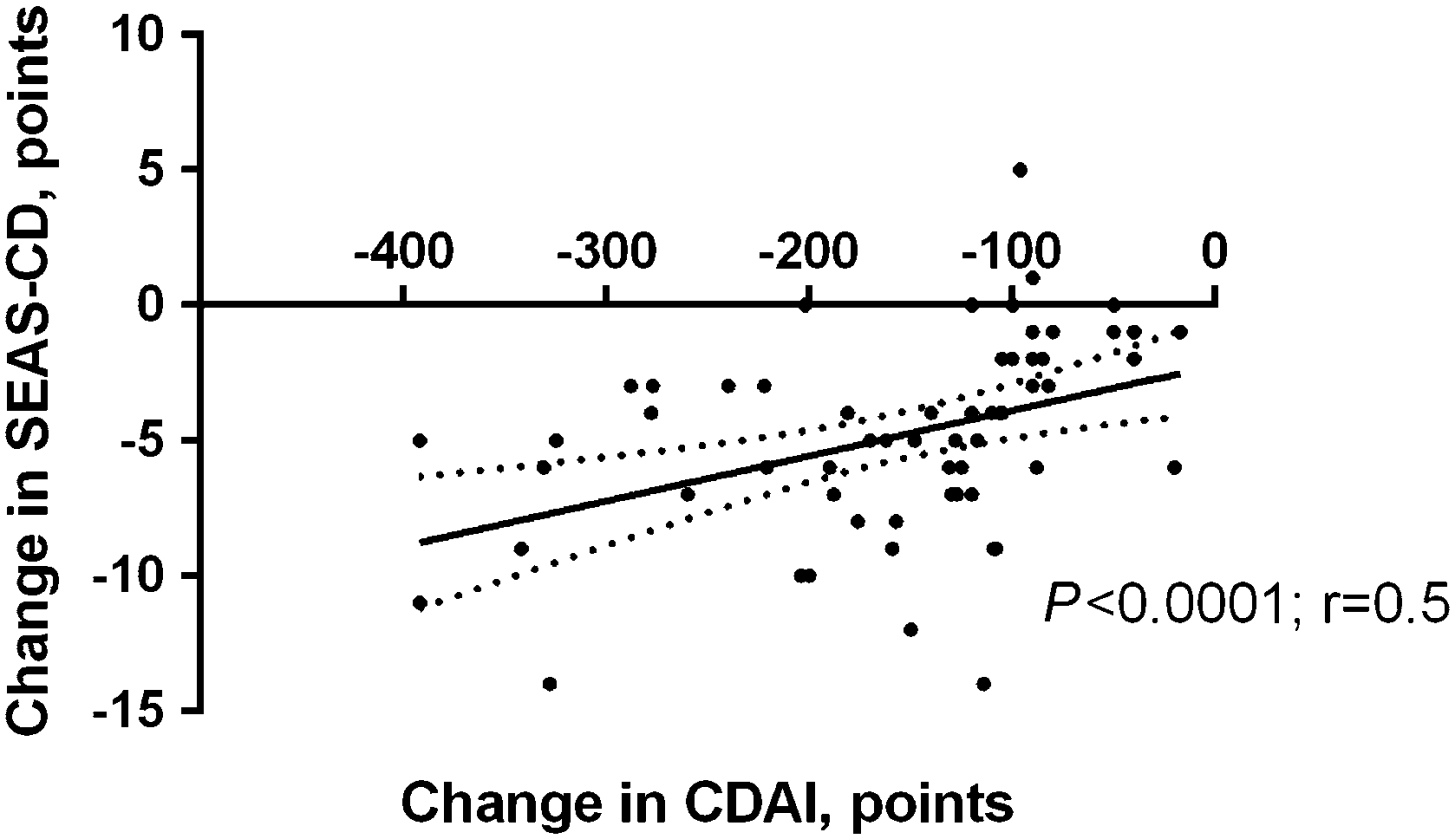
The correlation between the change in the Crohn’s Disease Activity Index (CDAI) and Simple Enterographic Activity Score for Crohn’s Disease (SEAS-CD) during the induction anti-tumor necrosis factor therapy.

53 patients (75%) were primary responders, whereas 18 (25%) did not respond to the induction doses of anti-TNF antibodies. The change in CDAI scores in the whole study group (*n* = 71) correlated significantly with fluctuations in SEAS-CD scores during induction anti-TNF therapy (Fig. 1).

**Fig. 2:**
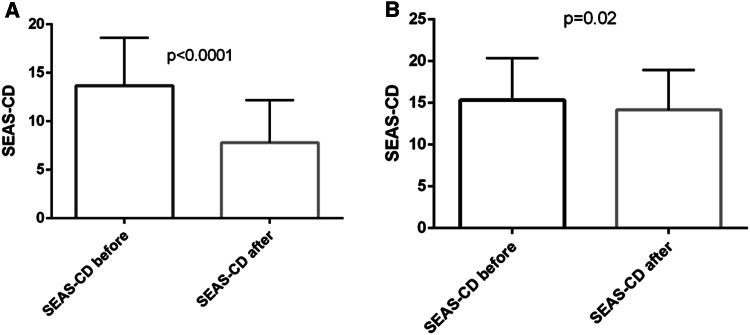
The change in the Simple Enterographic Activity Score for Crohn’s Disease (SEAS-CD) in the responders (**A**) and non-responders (**B**) group after induction anti-tumor necrosis factor therapy. Data are presented as means with standard deviations.

In the responders group there was a significant decrease in CDAI: 272 ± 90 vs. 94 ± 54 points (*P* < 0.0001), as well as a significant reduction of CD activity assessed in MRE—SEAS-CD decreased from 14 ± 5 to 8 ± 4 points (*P* < 0.0001) (Fig. 2A). We noted also a statistically significant change in high sensitive C-reactive protein (hsCRP) concentration—17.3 ± 25.6 vs. 3.6 ± 4.3 mg/l (*P* < 0.0001), hematocrit—37 ± 5 vs. 39 ± 5% (*P* = 0.01), hemoglobin concentration—12.2 ± 1.9 vs. 13.1 ± 1.8 g/dl (*P* = 0.001), platelet count—357 ± 105 vs. 302 ± 76 10^3^/mm^3^ (*P* < 0.0001), and erythrocyte sedimentation rate—27 ± 19 vs. 19 ± 17 mm/h (*P* = 0.001).

**Fig. 3:**
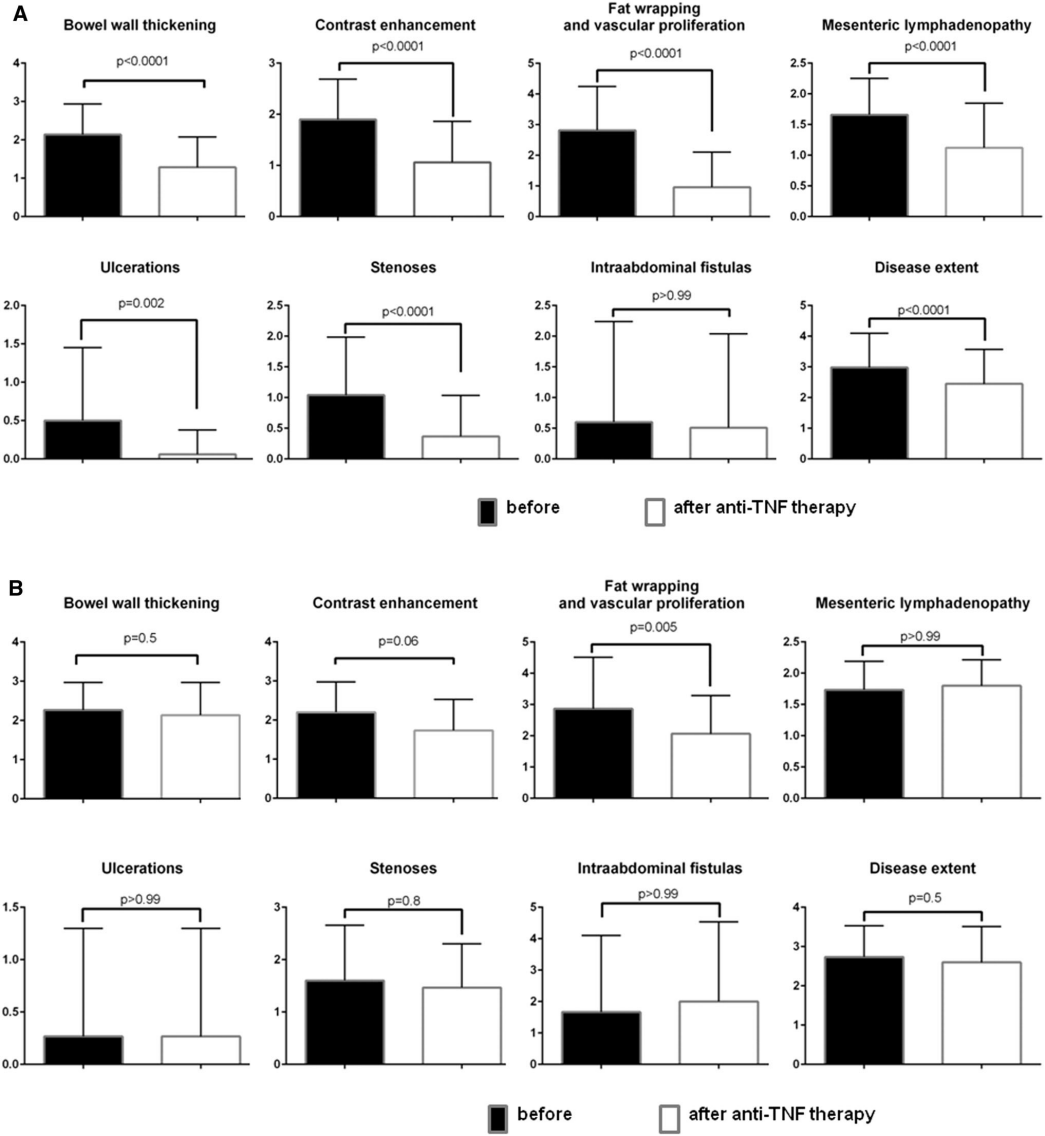
The change in the parameters of Crohn’s disease activity assessed in magnetic resonance enterography after induction anti-tumor necrosis factor alpha therapy in the responders group (**A**) and non-responders group (**B**). Data are presented as means with standard deviations.

**Fig. 4:**
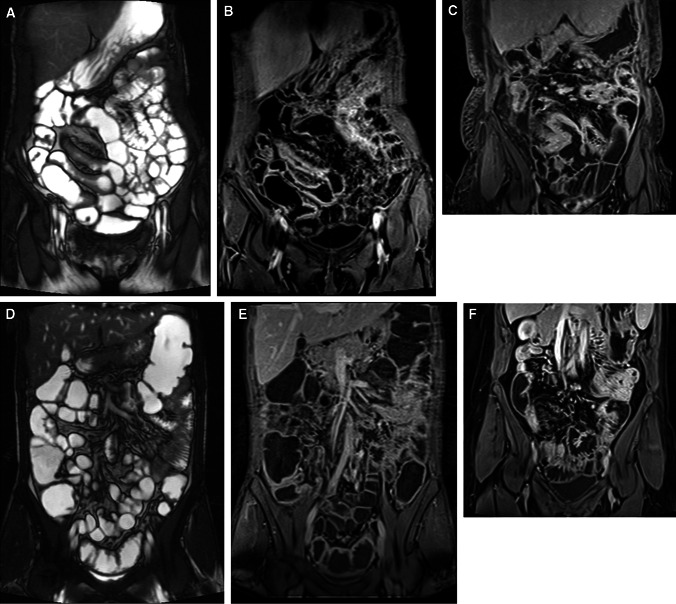
**A** T2-weighted sequence showing thickening of bowel wall before anti-tumor necrosis factor therapy (**A**). Dynamic contrast enhanced T1-volume interpolated gradient-echo sequence showing thickening of the bowel wall with layered enhancement, fat wrapping with a proliferation of mesenteric vasculature (**B**) and with enlargement of mesenteric lymph nodes before starting biological treatment (**C**). **B** T2-weighted sequence showing a significant decrease of bowel wall thickening after induction anti-tumor necrosis factor therapy (**D**). Dynamic contrast enhanced T1-volume interpolated gradient-echo sequence showing a significant decrease of bowel wall thickening without pathological enhancement, fat wrapping with a proliferation of mesenteric vasculature are not present after finishing induction biological treatment (**E**). The diameter of enlarged mesenteric lymph nodes decreased significantly after the therapy (**F**).

Almost all parameters of MRE CD activity decreased significantly after induction anti-TNF therapy in the responders group (Fig. 3A). Figure 4 shows examples of the influence of induction anti-TNF therapy on selected features of CD inflammatory activity seen in MRE among primary responders.

In the non-responders group CDAI did not change significantly after anti-TNF induction therapy: 275 ± 71 vs. 212 ± 77 points. Mean SEAS-CD values only slightly decreased in the course of biological therapy—15 ± 5 vs. 14 ± 5 points (Fig. 2B); however, taking into account the different distribution of variables before and after treatment, it reached the statistical significance (*P* = 0.02).

In the non-responders group, we also noted a statistically significant change in hsCRP concentration—27.1 ± 23.4 vs. 17.3 ± 27.7 mg/l (*P* = 0.04), platelet count—401 ± 130 vs. 349 ± 95 10^3^/mm^3^ (*P* = 0.01), and white blood cell count—6.5 ± 3.3 vs. 5.7 ± 2.7 10^3^/mm^3^ (*P* = 0.03). Other laboratory parameters did not change significantly.

There was a significant decrease only in fat wrapping and vascular proliferation after anti-TNF induction therapy in patients who did not respond to the treatment. Other parameters did not change or decreased without achieving statistical significance (Fig. 3B).

### Comparison of SEAS-CD between responders and non-responders group

Baseline SEAS-CD scores were not statistically different when compared the responders and non-responders group (14 ± 5 vs. 15 ± 5 points, respectively; *P* = 0.09). All assessed parameters of MRE CD activity were comparable between the two aforementioned groups.

**Fig. 5:**
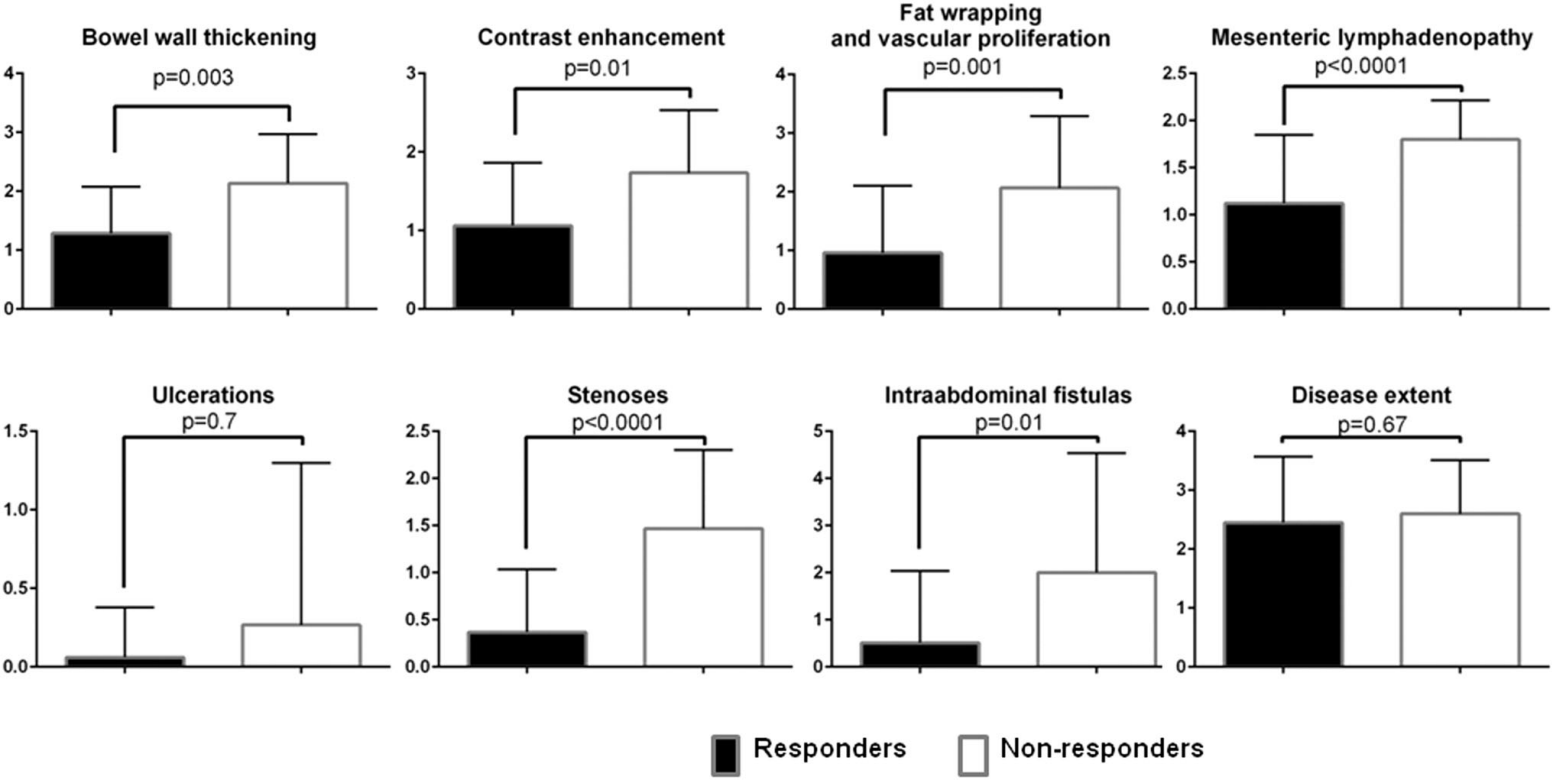
The differences in the variables assessed in magnetic resonance enterography after the induction phase of anti-tumor necrosis factor alpha therapy between the responders group and patients primarily not responding to treatment. Data are presented as means with standard deviations.

After finishing the induction phase of anti-TNF therapy, SEAS-CD among non-responders was significantly higher when compared with the responders group (14 ± 5 vs. 8 ± 4 points, respectively; *P* < 0.0001). Almost all parameters of MRE CD activity were significantly higher among non-responders after induction doses of anti-TNF antibodies (Fig. 5).

### One-year anti-TNF therapy

In 17 patients, MRE investigation was performed three times: at week 0, after anti-TNF induction therapy, and after 1 year of treatment. When compared CDAI and SEAS-CD scores after biological induction therapy and after 1 year of treatment, changes in those parameters correlated significantly (*P* = 0.004; *r* = 0.7). In this subgroup of patients—13 of them were in clinical remission (CDAI < 150) at week 52–54 of biological therapy; 4 patients were secondary non-responders (CDAI ≥ 150).

In patients with a long-term remission, there was a further, statistically significant improvement in CD MRE activity observed: SEAS-CD decreased from 9 ± 4 points after induction therapy to 5 ± 3 points at week 52–54 (*P* = 0.02). The decrease in SEAS-CD score was mainly due to further reduction in bowel wall thickening (*P* = 0.01), mesenteric lymphadenopathy (*P* = 0.03), and fat wrapping with vascular proliferation (*P* = 0.05, borderline significance). Other assessed MRE features did not change or improved without statistical significance.

In the subgroup of secondary non-responders, SEAS-CD increased from 6 ± 3 points to 10 ± 3 points; however, without statistical significance. All MRE features of CD activity assessed by SEAS-CD score increased after 1-year anti-TNF therapy without achieving statistical significance.

## Discussion

Objective assessment of the influence of anti-TNF therapy on the inflammatory activity of CD seems to be crucial for the proper monitoring of the treatment outcomes [9]. There are only few studies assessing the ability of magnetic resonance (MR) imaging techniques to determine the transmural and extraintestinal healing in CD patients treated with anti-TNF agents. First data in this subject concerned MR techniques that were similar, but methodologically different than MRE. Tielback et al. showed in their retrospective analysis including 50 CD patients that the inflammatory scores assessed in MR imaging improved only in individuals, who responded to the therapy [10]. Van Assche and colleagues performed a multicentre prospective trial, in which magnetic resonance enteroclysis was performed at baseline of anti-TNF treatment, and then at week 2 and 6 months after starting infliximab [11]. Finally, 15 patients were assessed at weeks 2 and 26. The analysis revealed that a decrease of the activity of inflammatory components occurred from 2 weeks after beginning of the therapy. At week 26, the magnetic resonance enteroclysis index improved in 80% of patients; however, only in 13% of them a complete absence of CD activity was noted.

In the most recent study performed by Ordas et al., the accuracy of MRE in determining the clinical response and mucosal healing in CD patients treated with anti-TNF agents were assessed for the first time [12]. This was a prospective multicentre study in which 48 patients were included, and they underwent ileocolonoscopy (as a reference standard) and MRE at baseline and 12 weeks after starting corticosteroids or anti-TNF therapy. The analysis revealed that the change in the MRE activity estimated with the use of Magnetic Resonance Index of Activity (MaRIA) determined with 90% accuracy the mucosal healing effect and with 83% accuracy—the endoscopic remission [13].

To the best of our knowledge, our study is the second one in which the utility of MRE in reflecting the influence of anti-TNF therapy on CD activity was assessed, and it includes the largest cohort of CD patients so far. Our analysis revealed that MRE CD activity changes in parallel with fluctuations of CD clinical activity during both—the induction (short-term) and 1-year (long-term) anti-TNF therapy. Almost all parameters assessed routinely in MRE are improving in patients clinically responding to the therapy in parallel to the majority of the laboratory results. Only intraabdominal fistulas were still present after treatment, what is to be expected, as this structural complication of CD is rather an indication for surgery [14]. However, in rare cases other therapeutic options are chosen, as not all patients accept surgery. The second and more frequent scenario is that patients underwent intestinal resection in the past and surgery has to be avoided in order to prevent irreversible complications after multiple intestinal resections, like for example, the short bowel syndrome. That is why some minor proportion of CD patients with intraabdominal fistulas is treated non-surgically, and anti-TNF therapy seems to be the most effective choice in these cases [14].

Although statistical analysis showed, that SEAS-CD decreased also in the non-responders group, this change was significantly less pronounced when compared with patients with CDAI-100 response. The decrease in SEAS-CD in patients not responding to the therapy was a consequence of a slight improvement in the minority of SEAS-CD variables; only fat wrapping with vascular proliferation decreased significantly. However, comparison of SEAS-CD scores after the induction anti-TNF therapy shows a significant and very pronounced difference between responders and non-responders group. Moreover, the majority of MRE variables differ significantly in these two groups of patients after the induction therapeutic period (Fig. 5), what shows that SEAS-CD reliably reflects the course of CD during biological therapy.

Nevertheless, considering only the non-responders group, anti-TNF treatment leads to transmural and extraintestinal healing to some extent, as well as there is also some improvement in single laboratory tests. However, these phenomena are insufficient to be reflected by regression of CD clinical activity. This is a very interesting phenomenon which was confirmed even on a molecular level. In the study by Leal et al. it was shown that also in patients not responding to the anti-TNF therapy, a big amount of inflammatory parameters involved in CD pathogenesis significantly decreased, although this was not reflected by the improvement of clinical status of the patients [15]. These data and our results suggest that CD symptomatology assessed by CDAI is influenced by multiple pathogenic factors, and only a complex, multifactorial improvement provides clinical response.

Analysis of the long-term influence of anti-TNF therapy also shows that changes in MRE CD activity parameters correlate with the clinical efficacy of biological agents. Comparative analysis shows that in the case of patients achieving a long-term response to the therapy, the degree of SEAS-CD reduction is significantly more pronounced during the first 3 months of treatment (induction therapy) than during maintenance anti-TNF treatment (mean change in SEAS-CD = −6 points vs −2 points, respectively; *P* = 0.0008). These data show that the strongest potential of anti-TNF agents to induce transmural and extraintestinal healing concerns the first, early period of treatment. Thus, it can be hypothesized, that the degree of the healing effect achieved during the induction anti-TNF therapy is a predictor of further treatment’s efficacy. However, it should be mentioned that even after 1 year of biological therapy, there was only one case of a complete recovery from inflammatory lesions seen in MRE (1/13–8% of patients in the long-term remission; data not shown).

In contrast to the induction anti-TNF therapy, which improved almost all MRE parameters of CD activity in the responders group, further clinically successive treatment results only in the significant decrease of bowel wall thickening, mesenteric lymphadenopathy, and fat wrapping with vascular proliferation; however, these data should be interpreted with caution due to a low number of patients experiencing the loss of response to anti-TNF agents. Nevertheless, it can be hypothesized that the aforementioned MRE parameters are mostly influenced by anti-TNF antibodies, and they are responsible to the greatest extent for transmural and extraintestinal healing phenomenon. The question that needs to be answered is whether these observations could be indirectly translated into hypotheses on the mechanisms and primary sites of action of anti-TNF agents. This could be very problematic, however, according to the latest reports on the pathogenesis of inflammatory lesions in CD, it is suggested that one of the crucial roles is played by the mesenteric adipose tissue in which significant vascular proliferation is seen [16]. It was hypothesized that so called creeping fat is a potent source of pro-inflammatory cytokines and other paracrine mediators, as well as it plays a significant role in the immune response to commensal bacteria [17]. In this hypothesis, further transmural inflammatory lesions are a consequence of an unusual transformation of mesenteric fatty tissue, as it was suggested that creeping fat in CD seen in MRE, has several features different from tissue seen in other gastrointestinal diseases, showing more inflammatory and fibrous pattern [18, 19].

According to these data, we observed that a significant influence of anti-TNF antibodies, seen in all study groups (short-term and long-term responders, as well as among primary non-responders), concerned only the mesenteric fatty tissue. Thus, one could hypothesize that the primary site of action of anti-TNF antibodies is the mesenteric creeping fat. Hypothetically, the decrease in the CD activity seen in MRE in terms of the mesenteric fatty tissue in the primary non-responders group could be an evidence, that anti-TNF antibodies started to act, but some poorly defined factors did not allow them to lead to further transmural and extraintestinal healing.

Our study has several limitations. In our opinion, the most important one is the retrospective nature of the analysis. However, as all MRE studies were blinded for the radiologist and then randomly analysed, we believe that this methodological fact reduces the risk of statistical bias. Another possible limitation is the use of concomitant medicines. Although the doses of immunosuppressive drugs were stable during the whole observation, tapering of steroids after finishing the induction phase of anti-TNF therapy or discontinuation of antibiotics were allowed in our study. It could be hypothesized that these facts can bias the interpretation of the direct influence of anti-TNF agents on CD inflammatory activity in MRE. However, in our real-life study group, we could not exclude those patients, as tapering of steroids or discontinuation of antibiotics is a consequence of successful application of anti-TNF antibodies. Moreover, steroids and antibiotics should not be used for a longer period of time. There are also no data regarding the intestinal healing effect of steroids or antibiotics in CD. This phenomenon has been shown only for anti-TNF agents and, to a lesser extent, for immunosuppressive drugs [9]. Thus, we hypothesize that the fact of tapering the steroids or discontinuing the antibiotics in our study group had limited or even had no influence on intestinal healing seen in MRE, and the observed phenomena in imaging studies are only related to application of anti-TNF antibodies.

Another possible limitation is the used SEAS-CD scoring system, as it assesses MRE features more on a global level and not on a bowel segment level, as it is done with the MaRIA system [12, 13]. This could be hypothetically problematic in patients in whom one intestinal segment responds to the therapy and other does not; however, such a situation is not likely to occur. Nevertheless, SEAS-CD takes into account the length of inflamed bowel loop, which is quantified and scored separately. Moreover, SEAS-CD scoring system assesses the CD activity (together with quantification of its extent) separately in the jejunum and ileum. That is why, although SEAS-CD does not allow for segmental assessment similar to MaRIA, it takes into account the different CD locations throughout the gastrointestinal tract and its extent in the bowel loops. Thus, it can be even reliable in the hypothetical aforementioned situation of simultaneous response and non-response to anti-TNF therapy in different bowel segments in the same patient.

To conclude, in our study, we showed that MRE is a useful tool in the assessment of CD course in patients treated with anti-TNF agents. We confirmed that transmural and extraintestinal healing occurs only in individuals responding to the therapy, although, the reduction of the inflammatory lesions seen in MRE takes place to some extent also in the non-responders. The most important compounds of the transmural and extraintestinal healing phenomenon seem to be the bowel wall thickening, mesenteric lymphadenopathy and wrapping of the mesenteric fatty tissue with the vascular proliferation. Data from our study cannot indicate the possible primary site of action of anti-TNF antibodies leading to intestinal healing, although the importance of mesenteric fatty tissue is discussed. There are too few proofs of the crucial role of creeping fat in the efficacy of biological therapy. We hypothesize, however, that our data could be an interesting background for further analyses on the role of mesenteric fatty tissue in anti-TNF therapy of CD.
